# How People with Physical Disabilities Can Obtain Social Support through Online Videos: A Qualitative Study in China

**DOI:** 10.3390/ijerph20032423

**Published:** 2023-01-30

**Authors:** Shiwei Huang, Yuwei Wang

**Affiliations:** School of Journalism and Communication, Jinan University, Guangzhou 510632, China

**Keywords:** online video creation, people with physical disabilities, social support, qualitative study

## Abstract

Background: Online video creation is becoming an option for many people with physical disabilities in China. However, few studies have considered how physically disabled individuals access social support through online video creation. Methods: For this qualitative study, semi-structured interviews were conducted with 18 online video creators with physical disabilities. The starting point of this study was video creation by people with physical disabilities, with a focus on the social interactions between creators, online video platforms, followers, viewers and sponsors, as well as the perceived social support in the process. Results: Thematic analysis was used to identify six social support themes: (i) meeting emotional needs; (ii) obtaining informational support; (iii) obtaining creative benefits; (iv) constructing identity; (v) reconstructing social relationships; and (vi) discovering self-worth. Four risk-related themes were identified: (i) online language violence; (ii) invasion of commercial capital; (iii) online video addiction; and (iv) fragile social support. Conclusions: The findings indicate that, although people with physical disabilities can obtain effective social support through creating online videos, it is accompanied by many risks. In addition, the social support gained through creating online videos differs from traditional online social support (e.g., changes in online support groups, emphasis on creative gain). Prospect: Future research should perform more detailed analyses of different types of social support and specific risks of creation, while taking into account individual differences and sociodemographic backgrounds.

## 1. Introduction

The China Disabled Persons’ Federation estimated that, at the end of 2010, approximately 85 million people in China had disabilities [1]. China has the largest disabled population of any country in the world. Research has estimated that the total number of disabled people in China will reach 165 million, which is almost double the current number, by 2050 [2]. The healthy development of such an enormous number of disabled people requires effective social support. Social support can positively affect self-esteem and self-efficacy, as well as promote many positive emotional, behavioral, and health outcomes across an individual’s life span [3].

Researchers have had high expectations of computer-mediated communication (CMC) as a means of providing social support [4]. While CMC scholars have produced a plethora of studies on different aspects of online behavior and outcomes, studies focusing on the online behavior of people with physical disabilities have only recently begun to emerge [5]. Indeed, online social support will change with developments in ways of socializing on the Internet [6].

The number of online video watchers in China had reached 995 million as of June 2022, which accounts for 94.6% of the total number of Chinese internet users [7]; considerable amounts of these videos are created by people with physical disabilities. At the macrolevel, the Chinese government’s disability policies are not based on a social model, but rather on an individual-oriented model, whereby disabled people must rely primarily on themselves rather than on the government [8]. This unique context prompts people with physical disabilities to actively use new communication technologies, such as online videos, for social support. In addition, past research has found that online interactions can help increase the level of perceived social support and improve people’s psychological state and health [5]. Social support for people with disabilities is not robust in developing countries, and emerging online technologies have the potential to enhance perceived social support for people with disabilities. In a broader sense, online video creation by people with physical disabilities can not only bring social support to themselves, but can also benefit a wider audience of viewers with disabilities and even able-bodied audiences. Therefore, it is worth exploring the behavior of people with physical disabilities in using online videos to obtain social support in the Chinese context. It should be further clarified that video creation by people with physical disabilities is only the starting point of this study; the social interactions between people with physical disabilities and online video platforms, followers, viewers, and sponsors, as well as the perceived social support in the process, are the focus of this study.

### 1.1. The Development of Online Social Support

Social support—“the various resources provided by one’s interpersonal ties [9]”—has been recognized by the health community as a means of promoting and restoring health and well-being [10]. Since the concept of social support was initially proposed, a large number of studies have aimed to reliably confirm the link between social support and physical health [11,12]. At present, sufficient evidence has shown that effective social support can promote recovery from various diseases [13].

With the rise of online networking, scholars have found that seeking social support is one of the most important reasons for people to socialize online [14]. A study found that social networking service (SNS) users feel higher levels of emotional support and companionship than ordinary internet users [15]. A growing number of scholars have conducted increasingly specific research on online social support. Many of these studies have focused on online social support groups on different platforms, such as WhatsApp and Facebook [16], Twitter [17], and Instagram [18]. Online groups can provide “just in time” support, overcome geographic barriers, and facilitate open discussion of health concerns [19]. At the same time, online video platforms have revolutionized ways of socializing and have brought new means of social support. Researchers have found that online videos provided a strong sense of presence and connection, even when viewed asynchronously [20], and that there was a high intensity of connection and emotion, thus opening up possibilities of new forms of online social support [21]. Gómez-Zúñiga and Fernandez-Luque [22] found that ePatients’ YouTube videos created a peer community for patients, met patients’ informational and emotional needs, and increased patients’ senses of self-efficacy. Tu and Zhou [23] found that young people with cancer received emotional care, identity reconstruction and self-worth, material rewards, and therapeutic assistance through TikTok short videos, thus transforming marginal support from strangers into core support in the real world. Salib et al. [21] pointed out that, while researchers have observed health videos on online video platforms, little is known about social support associated with online videos.

In addition, a growing number of studies have focused on online social support groups for specific conditions, such as postpartum depression [24], irritable bowel syndrome [25], Huntington’s disease [26], HIV/AIDS [27], breast cancer [28], Tourette syndrome, and tic disorders [29]. However, research on online social support for physical disabilities is insufficient [5]. Social support is even more important for populations with physical disabilities, because they are particularly dependent on the help of others. While individual experiences and circumstances vary widely, the reduced mobility, reduced socialization, and social isolation that can result from physical disabilities are common [30].

Although a variety of studies have investigated social support, most of them have been biased toward the structure or function of social support. Structural aspects of support are the perceived existence and number of support providers, whereas functional support refers to the perceived quality of support [31]. Researchers have argued that the measurement of functional support leads to a more relevant and valid representation of the quality and role of social support [9]. Most representatively, House [32] identified four forms of functional social support (emotional support, instrumental support, informational support, and appraisal support). These forms of functional social support are still being researched and expanded by scholars studying online social platforms, and the functional types of social support that act on perception play a more effective regulatory role for individuals [33]. Therefore, it is necessary to adopt a self-reported approach to social support for research [34].

### 1.2. Access to Online Videos by People with Disabilities

Recently, a growing number of people with disabilities have expressed various aspects of living with disabilities through video-based social media platforms that focus on media sharing [35]. When compared with traditional media technology, the potential of user-generated online videos lies in the opportunities it presents for online self-expression and exchange that is open, accessible, compelling, unconstrained, and unmediated [36]. Current research has focused on three aspects of the online video usage behavior of people with physical disabilities. First, some scholars have analyzed video content created by people with disabilities to investigate how these users present themselves and construct identities. Examples include discussing identity formation and identity management of people with disabilities in video game streaming [37], disclosures of self-difficulties by people with disabilities on YouTube [38], and visually impaired bloggers’ experiences in online video communities [39]. Second, some studies have noted the inclusivity of online video platforms for people with disabilities. Ta [40] made a normative case for why internet video creation should be accommodating of accessibility for people with disabilities and proposed how this ideal might be achieved with the inclusion of a road map of the technical, legal, and social hurdles that must be overcome. Rong et al. [41] found that TikTok has an algorithmic bias against blind or low-vision anchors and proposed that a more inclusive and fair live broadcast platform should be designed. Third, the use of online videos to obtain social support by people with disabilities has attracted the attention of researchers. Bromley [42] analyzed YouTube videos created by people with disabilities and argued that these videos function as informational support for other people with disabilities. Johnson [43] interviewed disabled anchors on Twitch.tv and found that live streaming brought them employment and money-making opportunities. Libin et al. [44] found that the popularity of “how-to” videos posted on YouTube by people with spinal injuries boosted these users’ senses of self-efficacy. Although online video platforms have become a key medium for people with disabilities to express themselves and obtain social support [35], few studies have discussed in depth how people with disabilities perceive social support through online video platforms.

In China, online video platforms such as Bilibili and TikTok have emerged with numerous users [45]. The popularity of online videos has inspired a large number of people with disabilities to create videos. According to media reports, many disabled people who have no access to traditional workplaces, due to time and space constraints, are finding employment by creating online videos [46]. Previous studies have pointed out that, under the dominant disability discourse of “kao ziji” (literally, relying on oneself) in China, disabled people lack direct support from the state; rather, the responsibility is left to the families of disabled people and to disabled people themselves [47]. However, China is currently undergoing a family structural transformation with a declining birth rate and aging population, which has made family risk expansion and family support fragility more obvious [48]. Therefore, disabled people in China value the social support provided by online video platforms. On this basis, it is worth studying the use of online videos by Chinese people with disabilities, especially those with physical disabilities, to obtain social support. Despite the urgent need to explore this area, there are few related studies.

Given that online video provides a new mode of communication and social support (based on the abovementioned literature) and that Chinese people with disabilities are actively using online video platforms to gain social support, it is important to understand how, for example, people with disabilities obtain social support through online videos. Evidence of the social support provided by China’s emerging online video platforms remains limited, so research on these platforms is warranted, as online video platforms such as YouTube are not easily accessible from within the country.

### 1.3. The Present Study

The abovementioned studies have provided some research evidence that people with disabilities are actively experimenting with online videos and that online videos can provide some social support for people with disabilities. However, there are some limitations: (i) most studies on online social support have focused on the dimension of structural social support, and research on perceptual social support needs to be strengthened; (ii) most relevant research has focused on social support on social platforms such as Twitter, but there has been little research on how people with disabilities use online videos to obtain social support; (iii) the only studies on the use of online videos by people with disabilities have focused on developed countries with relatively mature social support systems; however, social support systems for people with disabilities in many developing countries are still far from perfect, and how people with disabilities obtain social support through online videos has not received sufficient attention; (iv) previous studies on the use of online videos by people with disabilities have basically focused on the inclusiveness of digital technology or the identity construction of people with disabilities and have not taken social support as the main perspective; and (v) most relevant studies have focused on the positive effects of online videos on people with physical disabilities and have paid less attention to the possible negative effects.

In view of the fact that there are many classifications of disabled persons, for this study, physically disabled people were selected as the research object, and qualitative research was used to explore how physically disabled people use online videos to obtain social support without setting any research assumptions. As mentioned above, the social context and video platforms experienced by disabled people in China are special. This study is not aimed at a specific platform, but instead explores broader online video social support. The aim is to address the following questions: (i) In China, which is a developing country with an imperfect social support system for disabled people, why do people with physical disabilities choose online video creation to obtain social support? (ii) How do people with physical disabilities build social support through online video creation? (iii) What kind of social support do physically disabled people receive through online video creation, and what are the potential risks of this social support?

## 2. Materials and Methods

### 2.1. Study Design

A qualitative research design was used for this study. Qualitative inquiry can facilitate new areas of research by providing descriptive insights into the nature or meaning of everyday experiences [49]. Over the past few decades, there has been a clear qualitative methodological shift in disability research [50]. Attention has shifted away from the medical or rehabilitation definitions of disability as physical impairment or vocational restriction to a new definition of disability as a product of the interaction between the individual and the environment [51]. From this perspective, qualitative research can help to better disentangle the compounding effects of social, economic, and cultural barriers on overall health status and the health of people with disabilities [50]. Maxwell distinguishes between internal and external types of generalizations. The findings of qualitative research seek generalizability across settings or groups, without pursuing overly generalized external generalizations [52]. Therefore, this study did not formulate a research hypothesis, but rather examined an in-depth analysis of information on a specific topic. The semi-structured interview is a commonly used data collection method in qualitative research. The combination of semi-structured interviews and open-ended questions enables interviewers to navigate flexibly and explore the practices and feelings of people with physical disabilities who use online video creation to obtain social support, as well as allows new topics of conversation to arise [53]. The semi-structured interviews employed in this study were designed to investigate the reasons, processes, outcomes, and other perceptions of participants in obtaining social support through online video usage. The interview contained five main questions: (i) Why do you use online video platforms? (ii) How do you use online video platforms? (iii) Have the online video platforms brought you social support, and what kind of social support have they have brought? (iv) Has creating online videos effectively provided you with social support, and why? (v) What negative experiences have you had with online video creation?

### 2.2. Participants

Participants were screened in two rounds. First, DouYu (a Chinese webcasting platform), BiliBili (a Chinese video sharing platform), and TikTok (known as DouYin in China) were selected as representatives of China’s live broadcast, medium video, and short video platforms, respectively. The researchers selected six physically disabled creators from each of the three platforms, according to the criteria, for a total of 18 people. The criteria and process for selecting the interviewees were as follows: (i) search for creators with disabilities through the keyword “disability” on the platform; (ii) select creators with physical disabilities according to their personal profiles and content characteristics; and (iii) select creators with physical disabilities who update videos at least three times a week and rank among the top eight followers. Second, after the preliminary screening, the researchers contacted these 18 individuals through their video accounts, obtained their consent, and conducted semi-structured interviews with them. The interviews lasted between 30 min and 2 h and were fully recorded and transcribed. Pseudonyms are used to protect the anonymity of respondents, and their background information is shown in Table 1.

### 2.3. Ethics

All interviewees provided informed consent to participate in the study and were assured that their data were anonymous and confidential. Ethical approval for this study was obtained from the Institutional Review Board of Social Sciences and Humanities of Jinan University (IRB No. A2204001-025). This research was conducted in accordance with the Declaration of Helsinki. All data collection conformed with data protection regulations.

### 2.4. Data Analysis

Thematic analysis was used to analyze the qualitative data from the interviews. Thematic analysis is a method for analyzing qualitative data that entails searching across a data set to identify, analyze, and report repeated patterns [54]. Thematic analysis consists of six stages: (i) becoming familiar with the data (reading through all texts and becoming familiar with the main ideas); (ii) generating initial codes (coding features of the data systematically across the entire data set and collating the data relevant to each code); (iii) searching for themes (collating codes into potential themes); (iv) reviewing themes (checking and refining themes by reading all the data); (v) defining and naming themes (the research team discusses and agrees on the refinement of the theme names); and (vi) producing the report (selecting typical excerpts for presentation and writing a research report). It should be noted that, in order to eliminate researcher bias and prejudice, the authors invited a sociology professor of sociology and a graduate student of journalism and communication, both with a good research trajectory, to analyze together. Analytical rigor was ensured by the analysts scrutinizing, comparing, and discussing the coding to resolve any discrepancies identified. The agreement on the content analysis was assessed and was high [55]. Computer-assisted qualitative data analysis software (CAQDAS) was used, and the interview transcripts were input into NVivo Version 11 (QRS International, Melbourne, Australia).

## 3. Results

Two major themes were identified through thematic analysis: (i) social support for online video creators with physical disabilities and (ii) risks of social support for online video creators with physical disabilities. The first theme focused on why and how people with physical disabilities gain social support through online video creation, and the second theme revolved around the hidden risks for people with physical disabilities in gaining social support through online video creation. Under each major theme are subthemes expressing meaning and attributes. The subordinate themes of theme one are (i) meeting emotional needs; (ii) obtaining informational support; (iii) obtaining creative benefits; (iv) constructing identity; (v) reconstructing social relationships; and (vi) discovering self-worth. The subordinate themes of theme two are (i) online language violence; (ii) invasion of commercial capital; (iii) online video addiction; and (iv) fragile social support. Given that this was a qualitative study, the exact numbers were not necessarily important, since qualitative research does not attempt to generalize the findings. However, words are sometimes used in this study to indicate approximate numbers: “most” (more than 14 participants); “many” (9–14 participants); “some” (4–8 participants); and “a few” (fewer than 4 participants).

### 3.1. Social Support for Online Video Creators with Physical Disabilities

#### 3.1.1. Meeting Emotional Needs

Due to their physical impairments, people with physical disabilities are prone to negative emotions, such as anxiety and depression [56]. Online videos are easy to create and provide them with a channel to record and express themselves, which can meet their emotional needs to a certain extent.

All participants mentioned in the interviews that creating online videos provided them with a sense of companionship that they lacked in real life.

*“It’s not easy to take care of family members. They have their own things to do, and I don’t want to pass on some of my bad emotions to them. So I will use TikTok to record some of my emotions. TikTok can record with one click for short videos, and I use TikTok as a daily recording tool. When I tell the joys and sorrows in my life, I will receive some likes and comments, and I feel as if someone will always be with me.”* (M5)

*“My legs can’t move, and especially during the lockdown period, I can’t go out, so I often feel very lonely. But fortunately, I have become a TikTok anchor, although I only have a few thousand fans, but in the live broadcast room, there is always an audience listening to my story, which makes me feel a sense of companionship.”* (M16)

By creating short online videos, people with physical disabilities can be seen by more people. While watching the videos, viewers encourage and express caring for the physically disabled creators through Danmu comments (comments scrolling on the screen while an online video plays) and private messages. Most of the interviewees (16 out of 18) mentioned these comments.

*“I often share videos of me dancing with prosthetics on TikTok. At first, I thought that people would be afraid to see the crippled body, but then I found that many people encouraged me and praised me for dancing well. Almost every day, I see encouraging sentences in the comment section and private messages that make me feel warm.”* (M1)

*“Since the spinal injury, I have been paralyzed in bed, and life suddenly became very difficult for me. In order to allow myself to persevere. I started to share on BiliBili how to relearn life skills after being paralyzed. Many strangers encouraged me through Danmu comments, such as ‘We will always support you’ and ‘Come on, everything will be fine.’”* (M7)

#### 3.1.2. Obtaining Informational Support

People with physical disabilities usually undergo long-term rehabilitation, during which they need considerable social informational support, in addition to professional informational support from doctors. The interviews revealed that people with physical disabilities can obtain effective informational support, both active and passive, when creating videos.

All interviewees indicated that they shared their experiences and knowledge related to disability in their video creations. Before becoming video creators, they had usually collected much relevant health care and rehabilitation information online, thus ensuring their own knowledge. All participants mentioned proactive knowledge gathering for the purpose of creating online videos as an important motivation to obtain informational support for themselves.

*“In order to become a qualified short video creator, I watched almost all videos related to lower-limb disabilities on the Jitterbug platform. During this time, I also went to specialized rehabilitation websites for people with disabilities. Despite all my own years of experience, I was still worried that the rehabilitation methods I shared were not scientific and would mislead the audience. In the process, I grew into a half expert myself.”* (M2)

*“I usually watch videos on spinal injury rehabilitation and speak this knowledge to the viewers during the broadcast. In the process of learning and explaining, I not only feel that my knowledge about the subject has grown, but I also feel that I can better understand and manage my health condition.”* (M18)

Many participants (9 out of 18) indicated that online information was not reliable and that informed viewers added to or corrected the information they shared through comments. Of course, viewers with specialized knowledge backgrounds sometimes responded to the online videos and offered targeted advice to the video creators. In this ongoing interaction, the information provided by viewers served to support the creators of videos on physical disabilities.

*“When I was preparing to buy a prosthetic, I saw some information online about prosthetic reviews and shared it in a video. Later, several viewers who had been wearing prosthetics for years left me messages telling me that the information was actually advertising and marketing and recommended reliable information for me to buy prosthetics.”* (M1)

*“I was sharing my experience of studying counseling in the video and happened to meet a viewer who was a counselor. He shared a whole audio course on learning about counseling with me in his message, and I listen to one every day.”* (M7)

#### 3.1.3. Obtaining Creative Benefits

Participants also described how they earned income by creating online videos. All of the interviewees indicated that they were discriminated against when seeking work because of their physical disabilities, but that online video creation provided them with the possibility of earning money. The interviewees’ initial motivations for creating online videos were the desire to be paid, but only nine interviewees actually admitted to earning some income.

Five interviewees said that they had obtained a high number of fans and views through continuous online video creation. Then, webcast companies found them and trained them to be professional anchors.

*“I have searched for a job more than a dozen times. Every time the company sees my mutilated lower limbs, they will doubt my ability to work. I am good at playing games, and then I try to be a game anchor. Anyway, in the game live broadcast, the audience couldn’t see my legs. After the number of fans exceeded 30,000, a live broadcast company contacted me. I officially got a job as a game anchor and tasted the joy of working and making money.”* (M17)

*“In 2019, when live webcasting with goods had just emerged, I joined it with the mindset of trying it out. I was not professional at first and had several thousand fans watching, but the volume of trading was low. Later, a live-streaming platform recruited me and trained me professionally, and only then did the revenue slowly improve.”* (M2)

Four interviewees considered their ongoing online video creation to be a freelance job. Although the income was not high, it basically met their living needs. The numbers of likes and views on the online video platform did not provide much income, and the income obtained by the interviewees mainly came from rewards and donations from the audience after watching the videos.

*“I tried to work in a restaurant kitchen before, but the work was too intense and my body couldn’t support it. In comparison, I think online video creation is very freeing, and I can shoot with a mobile phone. I don’t have many fans, but every time, after sharing my daily life, a few fans will reward me.”* (M5)

*“I will post some popular songs that I cover on BiliBili, and fans who like me will join the corresponding fan group. Some fans will send red envelopes in the group to support me, and sometimes they even initiate donations to support my activity.”* (M9)

#### 3.1.4. Constructing Identity

Another theme that emerged from the qualitative analysis of the interview data was the construction of identity. All interviewees agreed that online video creation facilitated their self-identity. Previously, the identities of people with physical disabilities were shaped by mass media and other media, and these images were often distorted. Through online video creation, people with physical disabilities could take the initiative to present their image and, to a certain extent, break these social stereotypes.

*“For a long time, social culture has called us useless people, thinking that we are incapable, mentally retarded, and have weird behaviors. In fact, we are only physically disabled, not social waste. On BiliBili, I am a food blogger. I boldly admit that I am a disabled person, taste all kinds of delicacies, and let the audience see a disabled person who loves life, not a useless person who is desperate.”* (M10)

*“I grew up under the strange eyes of others, and many media and novels also vilify dwarfism. I used to feel that fate is unfair, and I would rather die than live. Later, I saw that there are actually many people like me on the Internet, and they are all struggling to live. So I tried to record my daily life in the webcast and challenge myself to do things that I never dared to do before. I know, I’m getting used to showing myself and accepting myself in the studio.”* (M14)

It is worth noting that three interviewees mentioned the role of official media in constructing the identity of physically disabled people. The official media reposted some of their videos, which aroused widespread praise on the Internet and brought them a large number of fans. The interviewees said that this represented their recognition by official media, which not only helped to reshape society’s perception of physically disabled people but also increased the ability of physically disabled people to construct their own identities.

*“The local Disabled People’s Federation forwarded the video of me dancing and invited me to perform on the show on the National Day of Helping the Disabled, which helped me increase tens of thousands of fans. I think this is a kind of affirmation of me, and the surrounding friends seem to have changed their views on my identity because of this incident, from pitying me to praising me.”* (M1)

*“I didn’t expect that one day, my video would be reposted by national media, and they were all amazed at my experience of teaching myself counseling while paralyzed in bed. At this moment, I felt that I accepted who I am and showed more people that people with physical disabilities also have a purpose in life.”* (M7)

*“I feel that we are living in a forgotten corner. The reports on me by the official media gave me hope. I know that I am being watched, and I begin to accept myself.”* (M13)

#### 3.1.5. Reconstructing Social Relationships

Participants also described the impact of creating online videos on their social relationships. Most participants believed that the low self-esteem associated with physical disabilities made them intentionally avoid social interaction and that online videos gave them the possibility of restructuring social relationships.

Two interviewees emphasized that creating online videos can build a virtual community for disabled people to eliminate the pressure of real social interaction.

*“Interacting with normal people made me feel stressed, and I would internally complain about why I was made disabled. But through video creation, I met a lot of people with physical disabilities just like me. When I talk and share with them, I feel that this should be the social relationship I need.”* (M3)

*“It’s hard to see people with physical disabilities in real life, and they prefer to stay at home. But the online videos I create provide a virtual gathering space where we can socialize without leaving our homes.”* (M12)

Most respondents believed that online social relationships established through online video creation can be translated into offline relationships.

*“I am very lucky. It is impossible to have so much dwarfism in real life, and it is impossible for me to find a normal person to marry. I created funny videos and met her, who also suffers from dwarfism, among the fans. Then, we developed from online to offline, and we got married after getting along for three months.”* (M6)

*“A fan left me a message after watching my video. She has the same lower-limb disability as me and lives in the same city. After we added WeChat to each other, we made an appointment to do rehabilitation training together. Now we have developed into good sisters.”* (M8)

Two other interviewees described the impact of online video creation on family relationships. The online social interactions that developed through video creation allowed them to come out of their self-imposed isolation and eased strained family relationships.

*“My parents were worried about my mental state, and they were afraid that I would self-harm or commit suicide because I couldn’t think about it. In the video creation, I met many people like me. I started to go out of my own world, and my parents were very relieved.”* (M4)

*“I have met many friends in video creation. I have become more cheerful, and my family no longer takes care of me all day long. I also tell my family funny stories about the process of creating the video, which provides us with more topics to talk about and enhances the relationship between my family and me.”* (M16)

#### 3.1.6. Discovering Self-Worth

Participants also reported discovering self-worth in the process of creating online videos. Most participants believed that online video creation provides opportunities for people with physical disabilities to learn knowledge and self-display, which contains the possibility of self-realization.

*“I not only create videos on the Internet, but I also use the Internet as a learning library. My video shooting, editing, and promotion methods are all learned online. I will summarize and apply various popular video creation skills. Through continuous learning, the number of views and fans of my videos have increased, and I think these data reflect my value.”* (M3)

One participant believed that self-worth is the ability to make money, and only when he could make money did he feel his own value.

*“In a market economy, money is valuable. I still can’t forget the first income that video creation brought me. I think I can finally make money with my own ability, and I am no longer a waste.”* (M5)

Other participants described feeling wanted because of the videos they created. Disabled video creators can find self-worth because of the needs of audiences, especially able-bodied audiences.

*“Most of my fans are actually able-bodied people who find the stories in my videos inspiring and bring strength to them. Sometimes, I still get private messages from fans thanking me for bringing them out of their slump with my videos. I feel like I am needed by everyone.”* (M15)

*“In the live broadcast room, many viewers admired how a disabled person is selling goods as soon as they came in. The audience may be attracted by my body at first, but then they will be infected by my content. I have some loyal fans, mostly able-bodied viewers, who make me feel included in society.”* (M2)

In addition, another interviewee talked about how creating videos changed his aesthetic vision. Creating videos requires the frequent discovery of beautiful things around him, which has changed his perception of himself and the world around him to a great extent.

*“Although my lower limbs are disabled, I am an outdoor anchor, and my purpose is to challenge myself. To shoot outdoor videos, you must have the ability to discover beautiful things around you while walking. The long-term shooting also trained my vision to discover the beauty of life. When I feel the world is interesting and beautiful, I feel like I’m shining too.”* (M8)

### 3.2. Risks of Social Support for Online Video Creators with Physical Disabilities

#### 3.2.1. Online Language Violence

When talking about the social support that comes with creating online videos, some participants repeatedly mentioned the negative emotional experience of online verbal violence. One participant reported in detail his experience of online verbal violence.

*“I am a food blogger, and the food is in great contrast to the dwarfism I suffer from, so when I first created the video, I often had to face language violence. Every time I open the message area, I always see comments accusing me of being ugly, polluting food, and begging on the Internet. For almost a year, I couldn’t get over my self-doubt.”* (M11)

Another participant spoke about the discrimination he experienced during the webcast.

*“One time I was randomly connecting with other anchors in the live broadcast room, but the other anchor entered the live broadcast room and saw my physical condition, said ‘It’s so unlucky, I’m mentally handicapped’ and left.”* (M18)

When asked how to deal with the negative emotions caused by this kind of online language violence, the participants commented that there is no good mitigation mechanism for this kind of negative emotion, and they can only rely on actively ignoring such comments, self-regulation, and comfort from other viewers.

*“We are not top creators, and the platform doesn’t care about us. When encountering this kind of language violence, it’s best to just ignore it. If I replied to every negative comment, then I would definitely be trapped in negativity and my life would be even grayer.”* (M2)

*There is also verbal violence in real life; just think more about happy things. If I encounter comments that attack me, I will simply delete them.”* (M9)

*“In fact, most of the viewers are very loving. When they see this situation, they will firmly support me and comfort me. Sometimes, they even help me to report these people.”* (M16)

#### 3.2.2. Invasion of Commercial Capital

Many participants (10 out of 18) spoke about the impact of commercial capital on video creation. They believed that, on the one hand, commercial capital provides rewards, and, on the other hand, it also creates troubles that cause them to feel alienated. One participant talked about his experience of being forced to make false sales for a merchant.

*“No one can make money from live broadcasting, let alone a disabled person like me. Later, an online store contacted me and asked me to help sell some products with quality problems, and I could share some profits from it. At that time, in order to make money quickly, I had to do this.”* (M2)

Some participants also said that commercial capital induces people with physical disabilities to create videos of miserable, ugly, and vulgar performances. This deviated from the original intention of the interviewees to reconstruct the identity of disabled people, but, in order to make money, they occasionally took the initiative to create related content.

*“I’m not really good at playing the game, but the audience thinks it’s awesome to be this way as a disabled person. Sometimes, in order to get the audience’s attention and reward, I will tell my disability experience over and over again and deliberately add some miserable episodes.”* (M17)

*“I’m a dwarf. Everyone thinks I’m ugly and deformed. So the media company I work for always asks me to play some ugly characters. Although I reject it in my heart, I understand that many viewers want to see my ugly appearance. Now, in order to make money, I began to take the initiative to sell ugliness.”* (M6)

Another participant described the guidance of platform traffic and algorithms to creative content. If vulgar content that can bypass online censorship is added, the click-through rate of the video will be better, and the algorithm may recommend it to more users.

*“Many viewers watch female anchors actually to watch vulgar content that plays rubbish. I found that dressing sexy, doing some sexually suggestive actions and saying some sexually suggestive content will get more viewers to click. The algorithm will also add you to the tags of sexy girls, sexy dancers or disabled girls for pushing. But you have to master a set of unspoken rules to bypass online censorship or you will be blocked.”* (M1)

#### 3.2.3. Online Video Addiction

Some respondents (5 out of 18) used the word “addiction” to describe their creation of online videos and the consequences. In addition, many respondents (9 out of 18) described the risk of loss of control in terms of realistic social and physical rehabilitation.

For example, one interviewee believed that the convenience and speed of making friends in the live broadcast room had made him start to separate from real social interaction.

*“In the live broadcast room, I can always find someone who is willing to listen to me and have feedback. But in real life, it is not easy for someone like me to find a listener. I am becoming more and more enthusiastic about webcasting, and sometimes I even broadcast for more than 10 h in a row. But I also obviously feel that I am out of touch with the real social life, and this computer seems to trap me.”* (M13)

Two of the participants felt that creating the video took so much time that it interfered with their physical recovery.

*“Singing videos are difficult to make, and you have to sing and revise the sound repeatedly. Generally speaking, it takes at least 6 h to make a video with higher quality. Such a large amount of sedentary time compresses my recovery time.”* (M9)

*“I only have one hand to use, and it is difficult for me to make short videos. For example, others can type and edit with both hands, but I can only type on the keyboard with one hand. It basically takes a day to make a video, and the recovery exercises have been interrupted for a long time.”* (M12)

In addition, many respondents indicated that they are very concerned about the playback data of the videos they create. They report checking for new likes and comments every few minutes, making them feel as if they have been hijacked by the web feed. One interviewee described his own experience in detail.

*“As long as I release a new video, I will keep clicking in to check the likes and comments of the video. There are no new social updates, but I still can’t help but check it every few minutes, worrying about missing something. This kind of behavior made me feel out of control. I knew it was a waste of time, but it was hard to control myself.”* (M3)

#### 3.2.4. Fragile Social Support

A final theme reported by participants was the fragility of social support gained through online video creation. Most participants (16 out of 18) believed that online social support is uncertain and transient.

Three participants shared their concerns about the sustainability of social support for creating videos, including factors such as personal age, content quality, and shifting online platforms.

*“I’m young now, and everyone likes to watch me sing and dance. But in a few years, when I get older, my fans may not like me anymore. What should I do then?”* (M1)

*“I broadcast four times a week, but my stories are limited. Fans who just joined the live room may be curious about my story, but slowly, they will lose interest in me, and I can feel that I am losing my fans.”* (M4)

*“Every few years, we experience a major reshuffle of online live-streaming platforms. I used to be an anchor in Panda Live, and after this platform closed down, I came to DouYu. However, the most popular now is the short video platform represented by TikTok. I worry that the platform I live on will one day also be eliminated, and then my efforts in the past few years will be in vain.”* (M17)

Some participants compared online and offline social support, arguing that online social support is only a supplement to offline social support rather than a substitute.

*“Like me being bedridden for years, my family and friends need to take care of me, and their support is definitely the first thing. Online videos are, after all, virtual and of limited direct help to me. I just hope that I can open up a new space on my own by creating online videos.”* (M7)

*“Internet space is fluid, and there are far more people watching me than helping me. Maybe a few of them are infected by my emotions, and they will help me further. In contrast, family and friends provide more lasting help.”* (M12)

## 4. Discussion

A large number of people with physical disabilities are becoming creators of online videos, but little research to date has focused on how people with physical disabilities access social support through the creation of online videos. For this study, semi-structured interviews were conducted with physically disabled online video creators, and a thematic analysis of the interview results was conducted. Respondents reported six dimensions of social support perceived through online video creation, including (i) meeting emotional needs; (ii) obtaining informational support; (iii) obtaining creative benefits; (iv) constructing identity; (v) reconstructing social relationships; and (vi) discovering self-worth. At the same time, respondents reported perceived risks of social support during video creation, including (i) online language violence; (ii) invasion of commercial capital; (iii) online video addiction; and (iv) fragile social support. Although participants reported both positive and negative experiences with social support, overall, they all confirmed that creating online videos was effective in obtaining social support.

As is consistent with previous research findings, access to face-to-face social support may be problematic for people with disabilities, due to their physical condition, mobility, and communication limitations; thus, they turn to the Internet for social support [57]. The participants in this study believed that the lack of offline social support was the reason that they wanted to become online video creators and, thus, obtain supplemental social support. Moreover, there is no substitution between offline and online social support; they are complementary relationships.

Previous research has shown that people with disabilities can use social media to create support groups [58], find experts on specific health issues [59], develop identity [60], gain knowledge [61], share emotions [62], and build friendships [63]. While previous research has focused on social support on social media platforms, this study extends the focus from social media platforms to online video platforms, thus confirming that people with physical disabilities can meet their emotional needs, gain informational support, construct identity, and reconstruct social relationships through creating online videos. In addition, three valuable findings were obtained from this study. First, there is no conclusive academic opinion as to whether weak connections in social media lead to effective social support. A recent study noted that weak connections are considered less helpful for emotional support and informational support [64]. In this study, all interviewees were active online video creators with high levels of online involvement. They perceived offline social support to be deficient, and they sought online social support to compensate. This finding is inconsistent with previous studies about social support from weak connections, which have most likely ignored the impact of the reality of the situation of seeking social supporters and the participants’ level of online involvement. Of course, this finding needs to be further verified by subsequent quantitative studies. In addition, a previous study noted that synchronous interactions can lead to stronger community cohesion and emotional energy than asynchronous interactions [65]. Participants in this study also mentioned the differences in social support from synchronous versus asynchronous interactions in online video. However, participants did not specifically describe the differences between the two, which warrants further study. Second, previous studies of social support on the Web have identified the existence of support groups [66]. The participants in this study did not explicitly mention support groups, but they believed that pop-ups and messages in online videos actually functioned as support groups to some extent. As interviewees M1 and M7 said, “In online video creation, we can throw out our questions in a targeted way, and there are always viewers who will give replies in pop-ups and messages, just like posting and replying in the disability community before”. At the same time, while traditional online support groups tend to have a homogeneous group of patients [67], the findings of this study indicated through interviews that online video support groups tend to be composed of able-bodied individuals, rather than those with the same physical disabilities as the participants. Further data analysis revealed that the temporary social support groups constituted in the online videos transformed into long-term fan communities. For example, creators often announce how to join their fan communities in their videos in order to gain more sustained social support. Third, this study found that people with physical disabilities gained creative benefits and discovered self-worth by creating online videos, which is a type of social support that has been overlooked in most previous related studies.

The qualitative data analysis in this study showed that earning income from creating online videos is a very important form of social support for people with physical disabilities. All participants reported that even though they were not currently earning income from creating online videos, they still harbor the hope of being able to do so. This can be further explained by previous research on people with disabilities and employment. Earning income through work is a sign of inclusion and participation in society for people with disabilities [68]. However, the Chinese government has not actively protected the employment rights and related benefits of people with disabilities in law, instead leaving such responsibility entirely to disabled people themselves and their families [69]. Furthermore, the government’s encouragement of disabled people to use the Internet for entrepreneurship and employment is part of a neoliberal policy of shifting social risk from the state to the individual in the wave of “Internet + disability assistance” in China [47]. In fact, only nine respondents in this study explicitly stated that they had earned income from creating online videos. Therefore, the effects of entrepreneurship and employment through the creation of online videos by people with physical disabilities need to be further studied.

People with disabilities are often stigmatized by the mass media as “supercrips”, disadvantaged, or victims of illness [70]. An empirical study suggested that positive mass media coverage of people with disabilities enhances their self-identity, while negative coverage leads them to deny their disability identity [71]. As is consistent with previous research, the interviewees in this study also reported a significant impact of positive mass media coverage on their identities. However, they also repeatedly used increases in likes, plays, and followers to measure this influence, which suggests that the identity elicited by mass media may be supplementary to traffic realization. In addition, participants repeatedly used the term “official media” rather than “mass media”, which reflected their perceptions of the role of official media in legitimizing discourse in China. Previous studies have also pointed out the dominant role played by Chinese official media in the legitimization of discourse [72].

People with physical disabilities encounter risks associated with accessing social support through online video creation. As is consistent with previous research, this study found vulnerability to the verbal violence that abounds online. This study also identified online video addiction that may result from the creation of online videos by people with physical disabilities. Previous research related to online addiction has focused on online addiction and application-specific addiction in youth groups, and research on possible symptoms of online addiction in people with physical disabilities is lacking. Respondents in this study reported symptoms of addiction and their consequences during the creation of their online videos. Another valuable topic is the intrusion of commercial capital. Previous research has suggested that Chinese women with disabilities are using performative bodies in social media to gain public visibility and resist being marginalized and stigmatized [73]. However, participants in this study described their own experiences of perceived body alienation by companies, platform traffic, and algorithms in the process of creating videos. It is worth noting that physically disabled online video creators are not ignorant of these risks, but are able to detect and take certain measures to cope with the sense of loss of control, which is a dynamic game process that deserves further study.

### Strengths and Limitations

The present study has certain strengths in four areas. First, to the best of our knowledge, it is one of the first qualitative studies to explore access to social support for people with physical disabilities through the creation of online videos and could contribute unique value for future, more in-depth related research. Second, this study adds to the findings of previous research on access to online social support for people with disabilities, particularly by providing revealing reflections on social support in weak relationships and the development of online support groups. Third, this study indicates the importance of creative gain and official media coverage for people with physical disabilities to access social support, which is an issue that has been infrequently discussed in previous studies. Fourth, this study reveals the hidden risks in the creation of online videos by people with physical disabilities and identifies many topics that have been overlooked in previous studies, such as addiction to online videos by people with physical disabilities, the alienation of creators with disabilities by commercial capital, and strategic resistance to online risks by people with physical disabilities.

However, this study has some limitations. First, participants may have exaggerated or concealed certain behaviors or feelings related to creating online videos in the interviews. This issue needs to be addressed, even though the interviewers in this study had experience in conducting interviews for qualitative research. Second, surveys and psychometric instruments were not used in the present study. It is an exploratory study in which people with physical disabilities reported social support and its risks in creating online videos. Future research can take specific interventions to measure the effects of social support obtained by creators with physical disabilities. Participants reported social support and risk for different themes, but did not rank these themes in order of importance. A qualitative survey of a small sample is a necessary step towards conducting a large-scale survey effort, and future research with a large sample could determine the order of social support and risk for different themes. Third, because this study is exploratory in the field, creators with physical disabilities were not categorized by gender, age, degree of disability, and time spent creating online videos, which may have affected the social support they received. In addition, creators with different types of disabilities may obtain variable social support through online videos. The participants in this study were people with physical disabilities, and further research should to see if this result can be generalized to the disabled population.

## 5. Conclusions

This study used a qualitative design to explore access to social support through online video creation for people with physical disabilities in China. As is consistent with previous research on online social support, this study found that people with physical disabilities can access effective social support through the creation of online videos, but it is accompanied by numerous risks. Future research could use different research methods to further explore this topic. In addition, future research on online video creation and social support for people with physical disabilities needs to consider individual differences and sociodemographic backgrounds as well as a more detailed analysis of different types of social support and risks in online video creation.

## Figures and Tables

**Table 1:** Sociodemographic characteristics of the participants.

Pseudonym	Age	Gender	Disability	Creation Platform	Followers
M1	18	F	Single Lower-Limb Disability	TikTok	430,000
M2	21	F	Lower-Limbs Disability	TikTok	120,000
M3	25	M	Single Lower-Limb Disability	TikTok	96,000
M4	28	M	Upper-Limbs Disability	TikTok	71,000
M5	20	F	Lower-Limb Disability	TikTok	53,000
M6	45	M	Dwarfism	TikTok	114,000
M7	26	M	Spinal-Cord Injury	Bilibili	32,000
M8	24	F	Single Lower-Limb Disability	Bilibili	15,000
M9	22	F	Lower-Limbs Disability	Bilibili	12,000
M10	24	F	Single Lower-Limb Disability	Bilibili	13,000
M11	33	M	Dwarfism	Bilibili	10,000
M12	23	F	Single Upper-Limb Disability	Bilibili	9000
M13	19	F	Single Upper-Limb Disability	DouYu	180,000
M14	32	F	Dwarfism	DouYu	76,000
M15	35	M	Spinal-Cord Injury	DouYu	11,000
M16	29	F	Upper-Limbs Disability	DouYu	5000
M17	30	F	Single Lower-Limb Disability	DouYu	49,000
M18	23	M	Spinal-Cord Injury	DouYu	6000

## Data Availability

The data presented in this study are available upon request from the corresponding author. The data are not publicly available for reasons of privacy.
